# ERP Evidence for Co-Activation of English Words during Recognition of American Sign Language Signs

**DOI:** 10.3390/brainsci9060148

**Published:** 2019-06-21

**Authors:** Brittany Lee, Gabriela Meade, Katherine J. Midgley, Phillip J. Holcomb, Karen Emmorey

**Affiliations:** 1 Joint Doctoral Program in Language and Communicative Disorders, San Diego State University and University of California, San Diego, CA 92182, USA; meade.gabriela@gmail.com; 2 Department of Psychology, San Diego State University, San Diego, CA 92182, USA; kmidgley@sdsu.edu (K.J.M.); pjh@neurocoglabs.org (P.J.H.); 3 School of Speech, Language, and Hearing Sciences, San Diego State University, San Diego, CA 92182, USA; kemmorey@sdsu.edu

**Keywords:** language co-activation, bimodal bilingualism, American Sign Language, deaf, ERPs

## Abstract

Event-related potentials (ERPs) were used to investigate co-activation of English words during recognition of American Sign Language (ASL) signs. Deaf and hearing signers viewed pairs of ASL signs and judged their semantic relatedness. Half of the semantically unrelated signs had English translations that shared an orthographic and phonological rime (e.g., BAR–STAR) and half did not (e.g., NURSE–STAR). Classic N400 and behavioral semantic priming effects were observed in both groups. For hearing signers, targets in sign pairs with English rime translations elicited a smaller N400 compared to targets in pairs with unrelated English translations. In contrast, a reversed N400 effect was observed for deaf signers: target signs in English rime translation pairs elicited a larger N400 compared to targets in pairs with unrelated English translations. This reversed effect was overtaken by a later, more typical ERP priming effect for deaf signers who were aware of the manipulation. These findings provide evidence that implicit language co-activation in bimodal bilinguals is bidirectional. However, the distinct pattern of effects in deaf and hearing signers suggests that it may be modulated by differences in language proficiency and dominance as well as by asymmetric reliance on orthographic versus phonological representations.

## 1. Introduction

Much research on bilingualism is focused on how a bilingual’s two languages interact (e.g., [1,2]). A topic of particular interest is the extent to which one language is co-activated while the other is being processed. Studies of language co-activation reveal how bilingual language users organize and control their two languages. Although language co-activation has been well documented in unimodal bilinguals, i.e., users of two spoken languages (e.g., [3,4,5]), fewer studies have examined this topic in bimodal bilinguals, i.e., users of both a signed and spoken/written language (see [6] for a review). Here we use the term ‘bimodal bilingual’ to describe both deaf and hearing signers. Deaf bimodal bilinguals are fluent in a signed language, are literate in the written form of a spoken language, and may have varying degrees of fluency in the spoken form of that language. Hearing bimodal bilinguals are also proficient in a signed language and are fluent in both the spoken and written forms of a spoken language. Studies with bimodal bilinguals can address how language co-activation occurs across modalities and whether the processes involved are the same or different from those involved in unimodal language co-activation (see [7] for discussion). 

Studies with unimodal bilinguals provide evidence that a bilingual’s two languages are not processed independently. Both of a bilingual’s languages are explicitly involved in translation tasks (e.g., [3]), but co-activation also appears to occur implicitly when only one language is selected for a given task and there is no apparent reason to call upon the other language (e.g., [4,5,8]). Thierry and Wu [5] used event-related potentials (ERPs) to investigate this implicit language co-activation. In this study, Chinese-English bilinguals were presented with pairs of English words and asked to make a semantic judgment. Unbeknownst to the participants, half of the English word primes had Chinese translations that contained a character that was repeated in the Chinese translations of the target words. Although there was no behavioral effect of the Chinese translation character repetition, ERPs revealed an effect of language co-activation. Specifically, the ERP analyses revealed an N400 priming effect such that English targets elicited smaller negativities when their Chinese translations were related in form to the Chinese translations of the primes compared to when they were unrelated. Thus, even when performing tasks in a monolingual context, unimodal bilinguals appear to activate lexical and sub-lexical representations from their other language (see also [2,9]). Wu and Thierry [8] conducted another study with Chinese-English bilinguals to dissociate the effects of phonology and orthography. They used the same implicit priming paradigm, but this time the Chinese translations contained either a sound repetition or a spelling repetition. Again, they found no behavioral effects of implicit language co-activation in either condition. ERPs revealed N400 priming effects for sound repetition but no effects of spelling repetition. They concluded that when processing English words, Chinese-English bilinguals implicitly co-activate Chinese phonology but not Chinese orthography.

Like unimodal bilinguals, there is evidence that bimodal bilinguals activate one language while processing the other. Hearing bimodal bilinguals appear to automatically access signs during auditory word recognition [10,11,12], and a number of studies have used the same implicit priming paradigm as Thierry and Wu [5] to demonstrate that bimodal bilinguals activate signs during visual word recognition. For example, Morford et al. [13] conducted a behavioral study in which deaf signers read pairs of English words and made semantic judgments. Word pairs that were related in meaning (i.e., “yes” responses) were judged more quickly when the American Sign Language (ASL) translations were related in form. For instance, *bird* and *duck* are related in meaning, and their sign translations share the same location and movement. Word pairs that were not semantically related (i.e., “no” responses) were judged more slowly when the ASL translations were form related. For example, *movie* and *paper* are not related in meaning, but their sign translations share handshape and movement. Thus, the phonological relatedness of ASL translations influenced semantic decisions for English words even though there is no phonological overlap between English words and ASL signs and ASL was irrelevant to the task. Kubus et al. [14] partially replicated these findings with German and German Sign Language (DGS): they found interference effects (for “no” responses) for form-related DGS translations but did not find facilitation effects (for “yes” responses). Finally, using the same semantic judgment task, Villameriel et al. [12] found both facilitation and interference effects for Spanish word pairs with form-related translations in Spanish Sign Language for hearing bimodal bilinguals (both native and late signers). These results support the notion that both deaf and hearing bimodal bilinguals access signs when reading words. 

Although co-activation of form representations in the non-target language appeared to impact semantic decisions in the target language, behavioral studies alone cannot deduce the mechanisms by which this co-activation occurs. Building on the behavioral studies with bimodal bilinguals described above, Meade et al. [15] used ERPs to examine the time-course of processing for sign co-activation during word reading. This study also used the Thierry and Wu [5] paradigm. Deaf ASL signers read pairs of English words and made semantic relatedness judgments. Semantically unrelated word pairs were manipulated so that their sign translations were related in form (i.e., shared two out of three major phonological parameters of location, movement, and handshape) or not. The authors found different patterns of co-activation for deaf signers who reported being unaware of the ASL form manipulation and those who were aware of the manipulation: the former (implicit) group showed a reduced negative response in form-related pairs 300–500 ms following target onset (an N400 effect); the latter (explicit) group showed a similar pattern but much later, 700–900 ms following target onset. The authors claimed that these early and late effects corresponded to automatic lexico-semantic processing versus more strategic translation processes that arose once participants became aware of the hidden form manipulation.

A number of studies have considered how bimodal bilinguals co-activate signs during visual or spoken word recognition, but few studies have explored whether the implicit co-activation of signs and words is bidirectional. To our knowledge, only two studies have investigated whether co-activation occurs from explicitly presented signs to implicitly activated spoken words, and both are unpublished. Van Hell et al. [16] asked hearing users of Dutch and Sign Language of the Netherlands (NGT) to view pictures and signs and verify whether or not they matched. For trials in which the picture and sign did not match, some of the picture names shared spelling (and rhymed) with the Dutch translation of the sign. This manipulation resulted in slower reaction times when the sign and picture had translations that overlapped orthographically and phonologically in Dutch compared to those that did not. The fact that spoken language overlap interfered with the verification task is evidence that these bilingual participants were co-activating their spoken/written language while performing a task with signed stimuli, even though the task did not require translation. 

Paralleling the behavioral results observed by Van Hell et al. [16], an ERP study by Hosemann [17] found evidence for co-activation of German translations when deaf native DGS signers viewed signed sentences. Each signed sentence contained a prime sign and a target sign whose German translations overlapped in orthography and phonology (e.g., LAST WEEK MY HOUSE, THERE MOUSE HIDE) (By convention signs are written in all capitals using the nearest translation equivalent). The EEG analysis indicated a smaller amplitude N400 to target signs preceded by related German translation primes versus those with unrelated primes. Although this priming effect is consistent with the idea that form-based representations of deaf signers’ spoken language are co-activated while processing their signed language, this particular study was limited by the fact that nearly half of the trials had to be rejected due to inconsistent translations. 

Despite recent progress regarding language co-activation in bimodal bilinguals, several questions remain unresolved: Is ASL-English language co-activation bidirectional? It appears that bimodal bilinguals co-activate signs during word recognition (e.g., [13,15]), but do they also co-activate words during sign recognition? If so, is the implicit co-activation of words the same for deaf and hearing bimodal bilinguals? In the present study, we used ERPs to address these questions and investigate co-activation of English words during recognition of ASL signs in the Thierry and Wu [5] paradigm. Deaf and hearing bimodal bilinguals viewed pairs of ASL signs and judged semantic relatedness. Half of the semantically unrelated signs had English translations that shared an orthographic and phonological rime (e.g., BAR–STAR) and half did not (e.g., NURSE–STAR). 

We predicted classic semantic priming effects (e.g., [18]) for both deaf and hearing signers, with faster responses and smaller amplitude N400s for semantically related trials (e.g., DOCTOR–SICK) compared to semantically unrelated trials (e.g., NURSE–STAR). We also hypothesized that ASL-English co-activation would be bidirectional and predicted that bimodal bilinguals would show evidence of co-activating words during sign recognition. All of the existing studies of implicit sign co-activation during word recognition in deaf signers resulted in behavioral interference effects (i.e., slower “no” responses to semantic decisions about words when their sign translations were related in form; e.g., [13,14,15]). We therefore predicted that deaf bimodal bilinguals would respond slower to semantically unrelated sign pairs when their English translations were related in form. There is also ERP evidence for N400 form priming effects in deaf signers, both when they co-activate signs during word recognition [15] and when they co-activate words during sign recognition [17]. Based on these findings, we predicted that targets in sign pairs with form-related English translations should elicit smaller N400s compared to targets in pairs with unrelated English translations. The two studies conducted with hearing signers showed the same pattern of behavioral interference whether they were co-activating signs during word recognition [12] or words during sign recognition [16], suggesting that hearing and deaf signers may show a similar pattern of behavioral and ERP effects.

On the other hand, there is reason to believe that deaf and hearing signers might differ in their co-activation of words during sign recognition, given differences in their language dominance and proficiency and in how they acquire English. For example, less proficient bilinguals tend to have stronger co-activation of their dominant language when completing tasks in their less dominant language compared to more proficient bilinguals (e.g., [19,20]). For hearing signers, English is their dominant language (see [7] for discussion), and thus they may exhibit greater co-activation of English when processing ASL (their less dominant language). Aside from language dominance, implicit co-activation may also be influenced by language proficiency. Both Morford et al. [13] and Meade et al. [15] found that the degree of ASL co-activation during word reading was correlated with reading ability (less-skilled deaf readers were more likely to co-activate ASL than skilled deaf readers). Hearing signers (even early-exposed signers) tend to be less proficient in ASL than deaf signers (e.g., [21,22]). Therefore, hearing signers with lower ASL proficiency may be more likely to co-active English during sign recognition. Finally, deaf individuals develop varying degrees of phonological knowledge depending on their language experience [23] but do not automatically access phonology when reading (e.g., [24]), which may affect how they develop and co-activate form-based representations of English words. Distinct patterns of processing for deaf and hearing signers could shed light on which linguistic factors influence how they co-activate words during sign recognition.

## 2. Materials and Methods

### 2.1. Participants 

Twenty-four severely-to-profoundly deaf bimodal bilinguals (mean age = 35.6 years; SD = 9.4) and twenty hearing bimodal bilinguals (mean age = 36.8 years; SD = 10.2) participated in this study. Deaf participants were all native or early signers of ASL (exposed before the age of seven) and reported ASL as their primary and preferred language. Hearing participants were highly skilled signers of ASL who had been signing for at least six years (mean number of years signing = 23.7 years; SD = 9.9). These participants varied in their age of ASL acquisition (mean = 12.2 years, SD = 8.5). Five participants were native signers (acquired ASL from birth), one was an early signer (acquired ASL before the age of seven), and 14 were late signers (acquired ASL after the age of seven, range = 10–24 years). Twelve participants reported that they work as ASL interpreters. Eighteen of the hearing signers rated their ASL skill as very good or advanced, one declined to report language background information, and one rated her ASL skill as good but completed the post-experiment translation task with high accuracy (over 80%; see Procedure below). One deaf signer and two hearing signers were left handed. All participants reported having normal or corrected-to-normal vision and provided informed consent in accordance with the Institutional Review Board at San Diego State University. Data from two additional deaf participants and one hearing participant were excluded from analyses due to blink artifacts contaminating a high proportion of trials (over 20%). One additional hearing participant was excluded from analyses because of very low accuracy on the post-experiment translation task (below 50%).

### 2.2. Stimuli 

Stimuli consisted of 188 pairs of ASL signs. Video stimuli were created by filming a deaf native signer producing each of the signs. The videos were then clipped two frames before sign onset and at sign offset in order to capture the phonological movement of the sign and remove transitional movements from the video. Sign onsets and offsets were determined following the same guidelines as in previous studies (e.g., [25,26]). A native signer and a fluent L2 signer independently determined the onsets and offsets for 20% of the stimuli and achieved high inter-rater reliability (98% agreement within a three-frame margin for sign onsets). The coders then split the remaining signs, with each coding onset and offset for an additional 40% of the stimuli.

Half of the ASL sign pairs (*N* = 94) were semantically related (e.g., LAUGH–HAPPY), and half of them were semantically unrelated (e.g., SHOES–HEAD). A norming study was conducted to determine how related the signs in each pair were. A group of 16 deaf participants read English glosses for each pair of signs and rated their semantic relatedness on a 7-point Likert scale (1 = totally unrelated, 7 = highly related). Sign pairs with average semantic relatedness ratings at or above 5.5 (mean = 6.36, SD = 0.35) were selected as stimuli for the semantically related condition, and sign pairs with an average rating at or below 2.5 (mean = 1.26, SD = 0.28) were selected as stimuli for the semantically unrelated condition. Semantic ratings were significantly higher for the semantically related condition than for the semantically unrelated condition, *t*(187) = 102.35, *p* < 0.001. 

**Figure 1:**
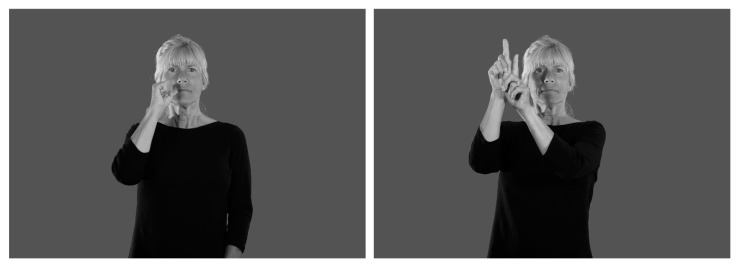
Example stimuli. A prime-target pair consisting of American Sign Language (ASL) signs with English rime translation (*bar–star*).

Half (*N* = 47) of the semantically unrelated pairs had translations that were form-related in English (e.g., BAR–STAR) (see Figure 1 for an illustration of these signs and see the Supplementary Materials Table S1 for a complete list of glosses for the ASL sign stimuli). Form-related pairs were defined as sharing both a phonological and orthographic rime. We use “rime” rather than “rhyme” to emphasize that the form overlap between the word pairs always occurred after the word onset and because the deaf participants are unlikely to be sensitive to sound-based rhymes. The other half (*N* = 47) of the semantically unrelated pairs did not share a rime (e.g., NURSE–STAR). To ensure that participants were co-activating English translations that did in fact share a rime, only signs with consistent translations were used. In a separate norming study, we asked 20 deaf ASL signers to view videos of ASL signs and provide their English translations. Only signs that were translated as the same English word by at least 16 out of 20 participants (80%) were included. Participants also completed a post-experiment translation task to ensure that their translations maintained the shared rimes (see Procedure). Signs in each pair did not share more than one phonological parameter in ASL (handshape, location, or movement). There was no significant difference between the semantic ratings for the rime condition (mean = 1.28, SD = 0.28) and the non-rime condition (mean = 1.24, SD = 0.29), *t*(93) = 0.50, *p* = 0.67.

Ideally, a full 2 × 2 design would similarly divide the semantically related trials into equal numbers of rime and non-rime pairs. However, it was only possible to find four semantically related sign pairs whose English translations also shared a rime (e.g., MAD–SAD). These form-related pairs were included to help prevent participants from developing a strategy that automatically equated form-related translation pairs with a “no” response in the semantic relatedness task. A debriefing questionnaire was also administered to determine if such a strategy was used or if the participants had noticed the form manipulation (see Procedure). Thus, the 94 semantically related pairs consisted of four rime translation pairs and 90 non-rime translation pairs.

### 2.3. Procedure 

During the experiment, participants were seated about 53 cm from the stimulus monitor in a dimly lit room. Deaf participants received instructions in both ASL and English, and a native deaf signer was present to answer any questions. Hearing participants received instructions in English. Participants were asked to view pairs of ASL signs and decide whether or not they were related in meaning. They used a video gamepad to respond as quickly and accurately as possible by pressing one button if the signs were related in meaning and a different button with the other hand if they were not. Response hand was counterbalanced across participants.

Each trial comprised a prime sign and a target sign with a 1300 ms stimulus onset asynchrony (SOA). Since the prime videos were variable in duration, a blank gray screen was added between the prime and target to maintain a constant SOA. Videos were presented on a black screen and subtended a visual angle of 10.8 degrees in the vertical direction and 14.0 degrees in the horizontal direction. Within the rectangular video frame, the sign model stood in front of a gray background and subtended a visual angle of 9.7 degrees in the vertical direction and 4.9 degrees in the horizontal direction. Following the target video, a black screen was displayed until 750 ms after the participant’s response. A purple fixation cross then appeared on the screen for 1500 ms, indicating to the participants that they could blink. A white fixation cross then appeared for 500 ms to indicate that the next trial was upcoming, and a blank screen was displayed for 500 ms before the next trial began. Participants were instructed to blink when the purple fixation cross was displayed and during longer breaks after every 12–15 trials. 

Each participant saw all 188 pairs of signs. Each target in the semantically unrelated pairs appeared once with an English form related prime (BAR–STAR) and once with an English unrelated prime (NURSE–STAR). When possible, targets in semantically related pairs also appeared once with an English form related prime (MAD–SAD) and once with an English unrelated prime (UPSET–SAD). However, due to the limited number of pairs that were related in both semantics and English form, most targets in semantically related pairs appeared with two primes whose English translations were unrelated in form (LUNG–HEART; BLOOD–HEART). Since each target appeared in the experiment twice, two pseudorandomized lists were created to minimize the effect of target repetition. For example, one list presented BAR–STAR in the first half of the experiment and NURSE–STAR in the second half, but the second list presented NURSE–STAR in the first half of the experiment and BAR–STAR in the second half. Lists were counterbalanced so that half of the participants in each group saw each list. The session began with 10 practice trials. The practice items did not contain any of the stimuli from the real experiment. 

Following the EEG portion of the experiment, participants were given a questionnaire that asked whether they noticed anything special about the sign pairs or whether they used any particular strategy to make their decisions. If so, we asked what they noticed, when they noticed it, and whether the pattern they noticed affected their ability to make semantic relatedness judgments. Fourteen deaf participants did not report noticing the form overlap in English translation rimes and were therefore included in an implicit co-activation subgroup. Ten deaf participants reported noticing the form overlap in the English translations and were included in an explicit translation subgroup. All but two of the hearing participants reported noticing the English translation rimes, so the hearing signers were not divided into implicit and explicit subgroups.

Following the debrief questionnaire, a translation task was also administered to participants to ensure that they produced the anticipated English translations. Any mistranslations that caused rime pairs to lose their shared orthographic and phonological properties were excluded from analyses on an individual basis (8 pairs, or 17.6% of rime pairs, for deaf signers; 12 pairs, or 26.1% of rime pairs, for hearing signers). So as not to differentially reduce the number of trials in the rime condition, the unrelated (non-rime) pairs with the same targets were also excluded from analyses. 

Several language assessments were also administered. The ASL Comprehension Test [22] and the long version (35 items) of the ASL Sentence Repetition Task [21] measure receptive and expressive skills in ASL, respectively. The Spelling Recognition Test [27] is a measure of orthographic precision in English. These measures of proficiency were collected for correlational analyses.

### 2.4. EEG Methods

Electrodes were placed on the participant’s left and right mastoids, below the left eye, and on the outer canthus of the right eye. The electrode on the left mastoid was used as a reference during recording and for subsequent analyses. The electrode below the left eye captured vertical eye movement and, along with recordings from FP1, identified blink artifacts. The electrode next to the right eye captured horizontal eye movement. Participants were then fitted with an elastic cap (Electro-Cap) with 29 active electrodes. Saline gel (Electro-Gel) was injected into electrodes to maintain impedances below 2.5 kΩ. EEG was amplified with SynAmsRT amplifiers (Neuroscan-Compumedics) with a bandpass of DC to 100 Hz and was sampled continuously at 500 Hz. 

**Figure 2:**
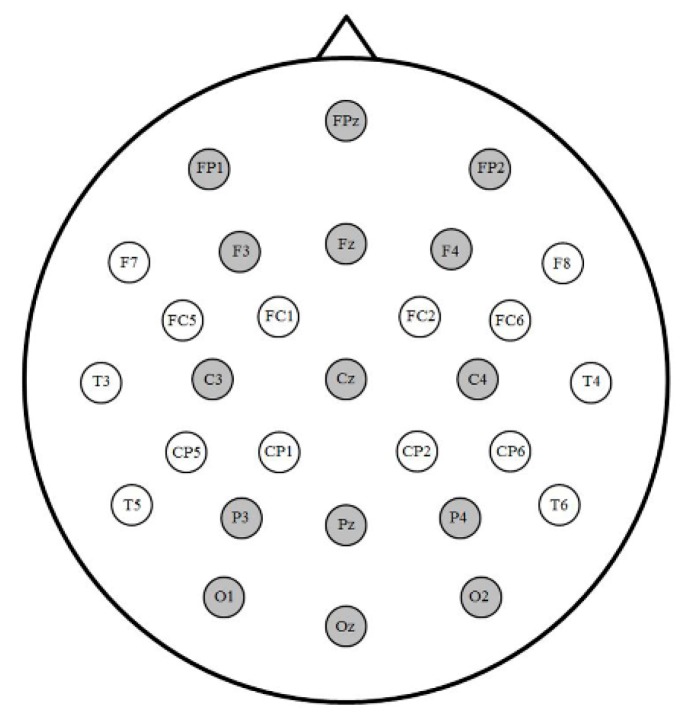
Electrode montage with gray channels included in event-related potential (ERP) analyses. Fifteen analyzed channels were distributed across five levels of Anterior/Posterior (Prefrontal, Frontal, Central, Parietal, Occipital) and three levels of Laterality (Left, Midline, Right).

Offline, ERPs were time-locked to target sign onset and averaged over a 1000 ms epoch, including a 100 ms pre-stimulus-onset baseline, with a 15 Hz low-pass filter at the 15 sites illustrated in Figure 2. Trials contaminated by artifacts from blinking, drift, or horizontal eye movement were excluded from analyses (5.2 trials, or 2.8%, on average for hearing signers; 7.1 trials, or 3.8%, on average for deaf signers). 

### 2.5. Data Analysis

Reaction times (RTs) were recorded for behavioral analyses. Linear mixed effects (LME) models with items and participants as random intercepts and participants as random slopes were used to analyze RT effects of semantic relatedness and rime overlap in the English translations.

Turning to the ERP analyses, mean N400 amplitude was measured between 325 and 625 ms. This window is consistent with our previous study of N400 effects using signed stimuli [26]. To analyze the N400 semantic priming effect, we used a mixed-design ANOVA with factors Semantics (Related, Unrelated), Laterality (Left, Midline, Right), Anterior/Posterior (Prefrontal, Frontal, Central, Parietal, Occipital), and Group (Deaf, Hearing), including only trials with correct semantic judgments. To analyze the N400 English translation effect, we used a mixed-design ANOVA with factors English translation (Rime, Non-Rime), Laterality (Left, Midline, Right), Anterior/Posterior (Prefrontal, Frontal, Central, Parietal, Occipital), and Group (Deaf, Hearing), including all semantically unrelated trials with correct translations. 

Meade et al. [15] also found evidence for a late effect (700–900 ms) of ASL co-activation when deaf signers were aware of the relationship among the ASL translations of the English word pairs they were reading. Thus, a mixed-design ANOVA with factors English (Rime, Non-Rime), Laterality (Left, Midline, Right), Anterior/Posterior (Prefrontal, Frontal, Central, Parietal, Occipital), and Group (Deaf, Hearing) was used to analyze mean amplitude within this late window. The semantic N400 effects, implicit English translation N400 effects, and later English translation effects were analyzed separately for the deaf and hearing groups as well as for the implicit and explicit deaf subgroups in planned comparisons. Greenhouse-Geisser corrections were applied to all ERP analyses with more than one degree of freedom in the numerator, and only significant effects are reported.

Finally, Pearson correlations were used to explore associations between the ERP effects and language proficiency in the deaf and hearing signers. ERP difference waves were calculated (mean amplitude of the unrelated–related conditions) for each of the 15 analyzed sites. Correlations were performed with the behavioral measures of ASL production, ASL comprehension, and English spelling for the N400 semantic effect, the N400 English translation rime effect, and the late (700–900 ms) English translation rime effect. Correlations were corrected for multiple comparisons using false discovery rate (FDR) corrections [28].

## 3. Results

### 3.1. Behavioral Results

Trials with incorrect responses were excluded from all behavioral analyses (12 trials, or 6.4%, on average for hearing participants; 10 trials, or 5.3%, for deaf participants). Trials with RTs shorter than 200 ms or longer than 2.5 standard deviations above each participant’s average RT were also discarded (4 trials, or 2.1%, on average in each group).

#### 3.1.1. Semantic Priming Effects

Mean RTs and error rates are presented in Table 1. The LME analysis of RTs indicated that deaf signers had faster responses overall compared to hearing signers, *t* = 3.25, 95% CI = [171.68,695.43]. The model also revealed a significant Group × Semantics interaction, with the hearing signers showing a larger semantic priming RT effect than the deaf signers, *t* = −2.43, 95% CI = [−189.99,−20.34]. Even though the semantic effect was larger in the hearing group, follow-up analyses confirmed a main effect of Semantics in both groups (deaf group, *t* = −8.18, 95% CI = [−142.13,−87.16], hearing group, *t* = −9.73, 95% CI = [−260.12,−172.88]). 

Within the deaf group, there was no Subgroup × Semantic interaction, meaning there was no difference in the size of the semantic priming RT effect for the implicit subgroup and the explicit subgroup, *t* = 0.72, 95% CI = [−95.30,44.03]. The implicit and explicit subgroups also had similar RTs overall, *t* = −0.09, 95% CI = [−186.25,205.03].

#### 3.1.2. English Rime Translation Effects

Only semantically unrelated trials were included in English translation analyses; half of these sign pairs had English translations that shared a rime and half did not. The LME analysis revealed a main effect of Group with deaf signers showing faster responses overall compared to hearing signers, *t* = 3.28, 95% CI = [145.56,577.72]. However, rime translations neither facilitated nor interfered with RTs, as there was no main effect of Rime, *t* = 1.00, 95% CI = [−40.22,123.21], or Rime × Group interaction, *t* = −0.88, 95% CI = [−71.25,27.02]. Within the deaf group, there was no difference in the size of the translation rime RT effect between the implicit subgroup and the explicit subgroup, *t* = 1.23, 95% CI = [−20.74,90.38]. Moreover, the implicit and explicit subgroups had similar RTs overall (main effect of sub-group: *t* = −0.47, 95% CI = [−228.90,140.78]).

### 3.2. ERP Results

Tables containing means and variances for each of the ERP effects reported below are available in the Supplementary Materials Table S2.

#### 3.2.1. Semantic Priming ERP Effect

##### Hearing vs. Deaf Signers

**Figure 3:**
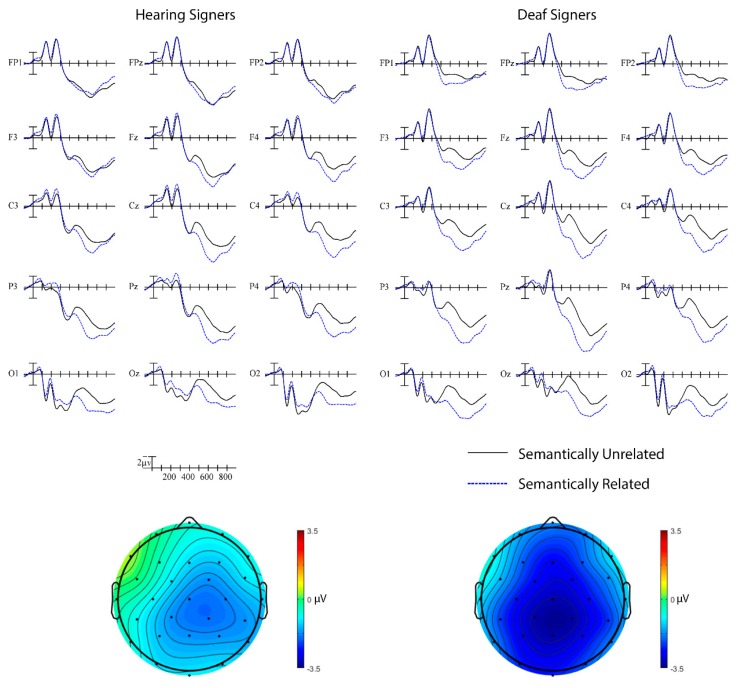
Semantic priming effects in hearing and deaf signers. Grand average ERP waveforms elicited by targets in semantically unrelated (black) and semantically related (blue) pairs at 15 sites. Each vertical tick marks 100 ms and negative is plotted up. The calibration bar marks 2 µV. Scalp voltage maps showing the semantic priming effect on mean N400 amplitude (unrelated–related) from 325–625 ms.

Semantics (325–625 ms). The semantic priming ERP results for the hearing and deaf signers are shown in Figure 3. The omnibus test for the semantic priming ERP effect yielded a main effect of Semantics, with semantically unrelated target signs producing greater negativities than semantically related trials especially over more midline and central-parietal sites, *F*(1,42) = 44.38, *p* < 0.001, η_p_^2^ = 0.51, Semantic × Anterior/Posterior × Laterality, *F*(8,336) = 5.95, *p* < 0.001, η_p_^2^ = 0.12. There was also a significant Group × Semantics interaction, *F*(1,42) = 5.78, *p* = 0.02, η_p_^2^ = 0.12, indicating that the N400 priming effect was stronger for deaf signers (M = −2.43 µV) than for hearing signers (M = −1.14 µV).

##### a. Hearing Signers

Semantics (325–625 ms). Within the hearing group, there was a clear N400 semantic priming effect such that semantically unrelated trials elicited larger N400s than semantically related trials, *F*(1,19) = 8.36, *p* = 0.01, η_p_^2^ = 0.31. This effect was strongest over right posterior sites, Semantics × Laterality × Anterior/Posterior, *F*(8,152) = 5.13, *p* < 0.001, η_p_^2^ = 0.21.

##### b. Deaf Signers 

Semantics (325–625 ms). Like the hearing group, the deaf group also showed a significant semantic priming effect, *F*(1,23) = 44.97, *p* < 0.001, η_p_^2^ = 0.66. For deaf signers, the effect was strongest over midline posterior sites, Semantics × Laterality × Anterior/Posterior, *F*(8,184) = 2.56, *p* = 0.04, η_p_^2^ = 0.10. This effect remained significant for both the implicit subgroup, Semantics, *F*(1,13) = 34.94, *p* < 0.001, η_p_^2^ = 0.73, Semantics × Laterality, *F*(1,13) = 4.33, *p* = 0.049, η_p_^2^ = 0.25, Semantics × Anterior/Posterior, *F*(1, 13) = 4.70, *p* = 0.02, η_p_^2^ = 0.27, and the explicit subgroup, Semantics, *F*(1,9) = 11.86, *p* = 0.01, η_p_^2^ = 0.57.

#### 3.2.2. English Translation Rime Priming Effect 

##### Hearing vs. Deaf Signers

**Figure 4:**
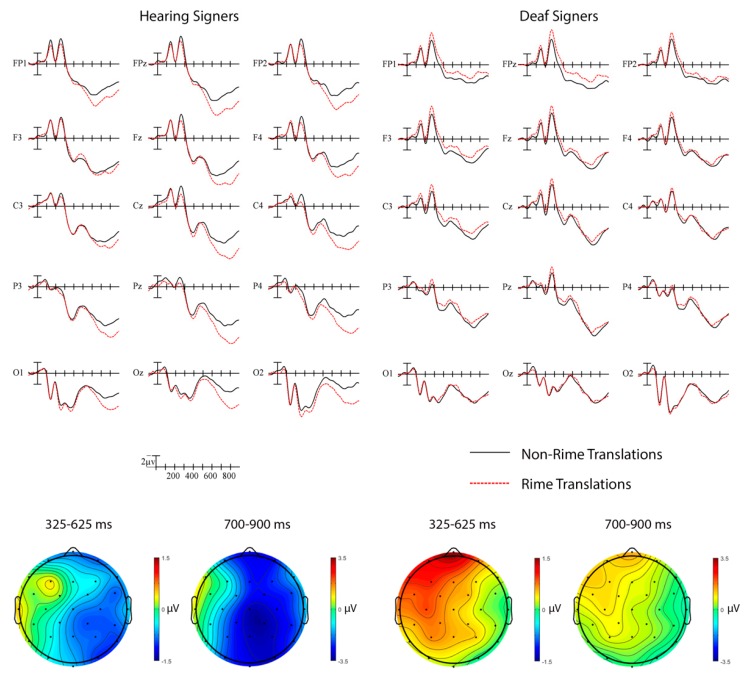
English translation rime priming effects in hearing and deaf signers. Grand average ERP waveforms elicited by targets in non-rime translation (black) and rime translation (red) pairs at 15 sites. Each vertical tick marks 100 ms and negative is plotted up. The calibration bar marks 2 µV. Scalp voltage maps showing the English translation priming effect on mean N400 amplitude (unrelated–related).

Rime (325–625 ms). The English translation rime priming results for hearing and deaf signers are shown in Figure 4. An omnibus ANOVA was conducted on the translation rime priming N400 ERP effect for deaf and hearing signers. A significant Group × Rime interaction indicated that the effect went in the standard priming direction (unrelated more negative than related) in the hearing signers (M = −0.53 μV) but in the opposite direction (related more negative than unrelated) in the deaf signers (M = 0.56 μV), *F*(1,42) = 4.71, *p* = 0.04, η_p_^2^ = 0.10.

Rime (700–900 ms). The omnibus test for the late effect of translation rime priming yielded a Group × Rime × Laterality interaction, *F*(2,84) = 9.58, *p* = 0.001, η_p_^2^ = 0.19. Targets in unrelated pairs elicited larger negativities compared those in related pairs, especially over right hemisphere electrodes, in the hearing group while in the deaf group it was the targets in related pairs that elicited larger negativities, especially over left hemisphere sites. 

##### a. Hearing Signers

Rime (325–625 ms). There was a significant English translation rime priming N400 effect in the hearing signers. Sign targets in pairs with non-rime English translations elicited larger negativities than sign targets in pairs with rime translations, especially at right hemisphere sites, Rime × Laterality, *F*(2,38) = 6.41, *p* = 0.01, η_p_^2^ = 0.25 (see Figure 4, left panel). 

Rime (700–900 ms). A large widespread English translation effect continued into the later window for hearing signers, Rime, *F*(1,19) = 11.41, *p* = 0.003, η_p_^2^ = 0.38, Rime × Laterality, *F*(2,38) = 15.96, *p* < 0.001, η_p_^2^ = 0.46, with greater negativities for targets with non-rime primes than for targets with rime primes, especially at right hemisphere sites (see Figure 4, left panel). 

##### b. Deaf Signers

Rime (325–625 ms). There was a significant reversed N400 effect of translation rime priming in the deaf signers. Sign targets in non-rime translation pairs elicited smaller negativities than targets in rime pairs within the N400 window, especially at left anterior sites, Rime × Laterality × Anterior/Posterior, *F*(8,184) = 3.24, *p* = 0.002, η_p_^2^ = 0.12 (see Figure 4, right panel). 

Rime (700–900 ms). Targets in non-rime translation pairs continued to elicit smaller negativities than targets in rime translation pairs in the later window, especially at left hemisphere sites, Rime × Laterality, *F*(2,46) = 7.56, *p* = 0.002, η_p_^2^ = 0.25 (see Figure 4, right panel).

##### b.1. Deaf Implicit Subgroup

**Figure 5:**
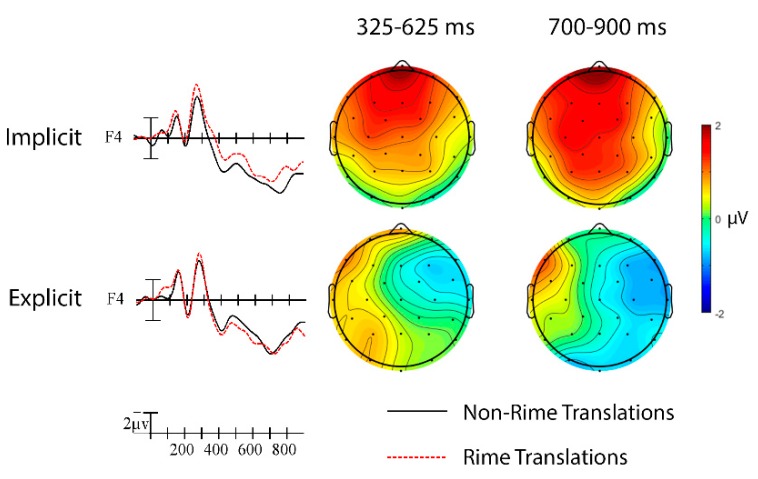
English translation rime effects in implicit and explicit subgroups of deaf signers at representative site F4. Scalp voltage maps showing the difference in mean amplitude between non-rime and rime translation trials for each of the analyzed time windows.

Rime (325–625 ms). The English rime translation results for the implicit and explicit deaf subgroups are shown in Figure 5. In separate analyses that included only the subgroup of deaf signers who were unaware of the English rime translation manipulation, we still observed the reversed N400 effect such that sign targets in non-rime translation pairs elicited smaller negativities than sign targets in rime translation pairs. This effect had the same left anterior distribution as in the whole deaf group, Rime × Laterality × Anterior/Posterior, *F*(8,104) = 2.98, *p* = 0.03, η_p_^2^ = 0.19 (see Figure 5, top). 

Rime (700–900 ms). For the implicit subgroup, sign targets in rime translation pairs continued to elicit larger negativities than targets in non-rime translation pairs in this later window, especially in the left hemisphere, Rime × Laterality, *F*(2,26) = 4.19, *p* = 0.04, η_p_^2^ = 0.24 (see Figure 5, top). 

##### b.2. Deaf Explicit Subgroup

Rime (325–625 ms). For the explicit subgroup of deaf signers who reported being aware of the English translation manipulation, the effect in the N400 window showed that the direction of the rime effect differed as a function of laterality, Rime × Laterality, *F*(2,18) = 4.71, *p* = 0.046, η_p_^2^ = 0.34 (see Figure 5, bottom). Sign targets in non-rime translation pairs elicited larger negativities than sign targets in rime translation pairs over right hemisphere sites (similar to the hearing signers), but smaller negativities than sign targets in rime translation pairs over left hemisphere sites (similar to the deaf implicit group). 

Rime (700–900 ms). The right-lateralized translation rime priming effect continued into the 700–900 ms epoch, Rime × Laterality, *F*(2,18) = 6.63, *p* = 0.01, η_p_^2^ = 0.42 (see Figure 5, bottom).

### 3.3. Correlations

Mean scores on each of the language assessments are reported in Table 2 for the hearing and deaf groups and the implicit and explicit deaf subgroups.

Correlations between these language measures and the ERP effects are reported below. All of the reported significant correlations were significant at multiple electrode sites. The site at which the correlation was strongest is reported below. See Supplementary Materials Table S3 for scatterplots of significant correlations at representative sites and Table S4 for a complete table of *r* values and corrected *p*-values for correlations across all sites. 

#### 3.3.1. Hearing Signers 

Semantic (325–625 ms). The amplitude of the semantic N400 priming effect was correlated with sign production ability. Less-skilled signers showed a larger semantic N400 effect at anterior sites (e.g., FP1), *r* = 0.61, *p* = 0.03. The correlations between the ERP semantic effect and the other ASL and English language measures were not significant, all *p*s > 0.20. 

Rime (325–625 ms). The English translation rime priming N400 effect was correlated with ASL comprehension test scores in hearing signers. Better sign comprehenders showed a larger N400 English translation rime priming effect at posterior and right hemisphere sites (e.g., Pz), *r* = −0.75, *p* < 0.001. The correlations between the N400 translation rime priming effect and the other ASL and English language measures were not significant, all *p*s > 0.25. 

Rime (700–900 ms). The late English translation rime priming ERP effect was also correlated with sign comprehension. Better sign comprehenders continued to show a larger translation priming effect at posterior sites (e.g., Pz), *r* = −0.63, *p* = 0.04. This late effect was also correlated with spelling ability. Better spellers exhibited a larger translation priming effect at posterior sites (e.g., P3), *r* = −0.67, *p* = 0.02. The correlation between the translation ERP effect and sign production was not significant, all *p*s > 0.96. 

#### 3.3.2. Deaf Signers

For deaf signers, none of the ERP translation or semantic effects were correlated with any of the language measures, all *p*s > 0.11. 

## 4. Discussion

This ERP study was one of the first to examine cross-modal, cross-linguistic co-activation of a written/spoken language when deaf and hearing bimodal bilinguals process a signed language. Participants viewed pairs of ASL signs and decided whether they were related in meaning or not. As predicted, we found behavioral and N400 semantic priming effects in both deaf and hearing signers. Half of the sign pairs that were unrelated in meaning contained a hidden manipulation such that their English translations shared orthographic and phonological rimes. We found evidence to suggest that both deaf and hearing signers do in fact co-activate English words during sign recognition, supporting the claim that ASL-English co-activation is bidirectional. Counter to our predictions, we found no behavioral effect of the hidden English form manipulation in either group. Despite the lack of behavioral evidence for English translation effects, we found the predicted N400 priming effect in hearing signers. However, we were surprised to find a reversed N400 effect of English translation for deaf signers. Analyses of implicit and explicit subgroups within the deaf group revealed that deaf signers had a reversed N400 effect regardless of whether or not they were aware of the hidden English form manipulation. For deaf signers who were unaware of the manipulation, this reversed effect persisted into the later 700–900 ms window. For deaf signers who reported being aware of the manipulation, the early reversed effect was overtaken by a later, more typical ERP priming effect similar to that found with the hearing signers. Thus, although ASL-English co-activation appears to be bidirectional, it manifests differently for deaf and hearing signers. 

Language dominance could account for some of the differences in effects between deaf and hearing signers. Hearing signers completed the task in their less dominant language (ASL), which could explain their slower RTs as well as the later and weaker semantic N400 effects compared to deaf signers. These differences in RTs and in the strength and latency of the N400 effect are consistent with studies of bilingual processing [29,30]. The semantic N400 priming effect was also correlated with signing ability for hearing signers, with less-skilled signers showing a larger semantic N400 effect and benefitting more from the semantic relationship between primes and targets than more skilled signers.

However, language dominance does not seem to explain the English translation effects. When deaf signers read English words in a previous study, ASL translation priming effects correlated with reading ability [15]. Less-skilled deaf readers co-activated ASL when reading English words to a greater extent than skilled deaf readers, which might aid in the processing of English, their less dominant language. Based on this pattern, we would expect the English translation priming effects in the hearing signers of the present study to correlate with signing skill such that signers who were less proficient in ASL (their less dominant language) would show greater co-activation of English (their dominant language). However, the reverse was true: better hearing signers showed stronger English translation priming effects for both the N400 and later windows than less-skilled hearing signers. One possible explanation for this pattern relates to the fact that the majority of the hearing participants (18/24) were professional ASL-English interpreters and the remaining participants were either training to be interpreters or had some experience as volunteer interpreters (e.g., for deaf family and friends). Experience with simultaneous interpreting could establish strong lexical connections between ASL signs and English words, such that more skilled signers activate the English translations of signs more robustly than less skilled signers.

Based on previous results, we expected slower RTs to targets in semantically unrelated pairs whose English translations were related in form compared to those that were not [12,13,14,15]. However, there were no behavioral effects of English translation in either group. One speculative explanation for the different findings is that the bidirectional links between words and signs are asymmetric, with stronger links from words to signs than from signs to words. Our findings also differ from Van Hell et al. [16], who reported slower RTs in a sign-picture verification task when translations were phonologically and orthographically related in Dutch. It is possible that pictures activate word forms more strongly than sign translations, such that the word form overlap interferes more with the sign-picture verification task than with the semantic relatedness task.

The absence of behavioral interference effects in the present study more closely resembles the findings in studies of unimodal bilinguals by Thierry and Wu [5] and Wu and Thierry [8]. Unlike most alphabetic scripts that conflate phonology and orthography, Chinese allows for independent manipulation of either sound or spelling. The hidden form manipulation in Wu and Thierry [8] used English word pairs whose Chinese translations either sounded alike (shared phonemes) or looked alike (shared graphemes). If the lack of behavioral effects was related to the weak correspondence between the spoken and printed forms of Chinese, perhaps a dissociation of phonology and orthography could also explain the lack of behavioral effects in the present study since ASL has no written form. In fact, Wu and Thierry [8] argued that cross-language N400 priming was due to implicit co-activation of phonology as opposed to orthography. 

Despite the lack of behavioral effects, we did find ERP evidence for co-activation of English translations that differed for hearing and deaf signers. Hearing signers likely accessed both the orthographic and phonological rimes of the English translations. As in the study by Wu and Thierry [8], the smaller N400 in the hearing signers was likely due to the phonological overlap of the English translation rimes more so than the orthographic overlap. The deaf signers did not show the N400 priming effect because they have reduced access to English phonology. 

Why, then, did deaf signers show any N400 effect at all? Since they are not likely to automatically access phonology [24,31,32], they may make use of alternative or supplementary approaches to reading. For example, deaf readers may build cross-language associations and improve vocabulary through methods like “chaining” [33]. With this instructional method, a teacher models a sign along with its corresponding fingerspelled word and its English translation in print. These successive multi-modal representations of the same concept strengthen associations between printed words, signs, and concepts. This difference in language acquisition may affect how deaf readers organize orthographic, phonological, and semantic information in their lexicon. More specifically, they may rely more heavily on orthography and direct orthographic-to-semantic connections (e.g., [34]). 

One speculative explanation for the reversed N400 seen in deaf participants, then, is that co-activation of orthographically similar words results in competition, rather than facilitation. Meade, Grainger et al. [35] found a reversed N400 priming effect among deaf and hearing readers; words preceded by orthographically similar prime words elicited larger amplitude N400s than those preceded by orthographically similar pseudowords. They interpreted this effect as an indication of lexical competition and inhibition between orthographic neighbors when the primes were real words. Presumably, hearing signers also experienced orthographic competition when they automatically co-activated orthographic representations in the present study, but the effect of phonological priming appears to have been so strong as to override it. Orthographic competition may be more apparent in deaf signers because they may rely more heavily on orthography (and less on phonology) for lexical access. 

The deaf participants who reported being aware of the hidden English manipulation are key to interpreting the results, as their English translation effects were reversed in the N400 window (like the implicit deaf signers) but went in the direction of priming in the later window (like hearing signers). Specifically, the early reversed effect may indicate that they were implicitly co-activating English orthography (leading to competition between orthographic neighbors), but the later priming effect could indicate that they explicitly activated phonology once they accessed the lexical representation and were aware of the manipulation. In support of this hypothesis, Cripps, McBride, and Forster [36] found a facilitative phonological effect for hearing readers and an inhibitory orthographic effect for deaf readers in a masked priming study. They argue that early orthographic processing is distinct from later phonological processing for deaf readers and attribute late priming effects to a “phonological post-access check.” The late priming effects seen in the hearing group and the explicit deaf subgroup in the present study could also be evidence for participants verifying the phonological representations generated top-down from the accessed lexical entry against those generated bottom-up from orthography. Indeed, the late English translation effect correlated with spelling skill for hearing signers and showed a trend in this direction for deaf signers. This relationship suggests that the late English translation priming effect is linked to orthographic precision and that orthography is checked against phonology at this later stage of processing.

Future studies should tease apart the role of orthography and phonology in language co-activation for bimodal bilinguals. However, since orthography and phonology are conflated in English, separate sound and spelling manipulations as in Wu and Thierry [8] may not be possible in studies with ASL-English bilinguals. A potential follow-up study could make use of phonological overlap with and without orthographic overlap (e.g., *dance-chance* versus *pants-chance*). This design could dissociate the different effects of co-activating orthography versus phonology for deaf and hearing ASL-English bilinguals. Alternatively, language co-activation could be studied in bimodal bilinguals who are literate in languages that use non-alphabetic scripts. Logographic characters like those used in Chinese and Japanese would allow for easier separation of orthographic and phonological effects.

## 5. Conclusions

In sum, this study suggests that implicit cross-language co-activation in bimodal bilinguals is bidirectional. However, co-activation of sign from print does not look the same as co-activation of print from sign in deaf bimodal bilinguals. We also noted differences between deaf and hearing bimodal bilinguals and between deaf signers with and without explicit knowledge of the hidden cross-language manipulation. These findings enhance our understanding of how bimodal bilinguals represent and use their two languages, which may be especially important for promoting literacy in the deaf population, and contribute to our knowledge of bilingualism overall.

## Figures and Tables

**Table 1:** Semantic and rime reaction times (mean (SD)).

Group	RT (ms)			
	Semantically Related	Semantically Unrelated	Rime	Non-Rime
Hearing Signers	1193 (243)	1383 (304)	1375 (295)	1392 (321)
Deaf Signers	1016 (176)	1132 (210)	1140 (208)	1123 (218)

**Table 2:** Language assessment scores for hearing and deaf signers (mean (SD)).

Group	English Spelling	English Reading	ASL Production	ASL Comprehension
Hearing (*N* = 20)	79.0 (3.5)	91.8 (5.8)	14.8 (5.1)	82.0 (5.1)
Deaf (*N* = 24)	74.4 (7.3)	79.6 (13.1)	22.6 (5.13)	86.5 (9.4)
Implicit (*N* = 14)	72.1 (6.9)	75.0 (14.3)	21.4 (5.8)	83.3 (10.8)
Explicit (*N* = 10)	77.7 (6.9)	86.1 (7.7)	24.3 (3.7)	91.1 (4.5)

## Supplementary Materials

The following are available online at https://www.mdpi.com/2076-3425/9/6/148/s1, Table S1: Glosses for ASL Video Stimuli, Table S2: Mean (SD) for ERP Effects by Group and Subgroup, Table S3: Scatterplots of Correlations between ERP Effects and Language Measures at Representative Sites, Table S4: Pearson Correlations with FDR-Adjusted *p* Values.
