# Supplementary material for: ERP Evidence for Co-Activation of English Words during Recognition of American Sign Language Signs
Source: Brain Sci. 2019 Jun 21;9(6):148. doi: 10.3390/brainsci9060148 (PMC6627215; doi:10.3390/brainsci9060148)
Supplement: Supplementary file 1 [file brainsci-09-00148-s001.zip › Table S1.pdf]

Supplementary Materials. Glosses for ASL Video Stimuli

Semantically Unrelated Prime-Target Pairs

	Non-Rime Prime	Rime Prime	Target
1	MY	HAT	BAT
2	RULE	GLASS	GRASS
3	SORRY	FINE	NINE
4	FLAG	FLOOR	POOR
5	SILLY	BALLOON	NOON
6	SHOES	HEAD	BREAD
7	USE	STUCK	DUCK
8	LAW	TREE	SEE
9	WHERE	FULL	BULL
10	EYE	PLAIN	TRAIN
11	NEVER	BELL	SPELL
12	HUNT	BLINK	PINK
13	WORK	LINE	WINE
14	ROOM	CLOCK	SOCK
15	SIGN	THIN	WIN
16	NOW	WOOD	HOOD
17	VOICE	BALL	FALL
18	WHY	CUT	NUT
19	BODY	COAT	GOAT
20	STAFF	BOX	FOX
21	AND	ARM	FARM
22	PIPE	THINK	WINK
23	OWL	SHAVE	SLAVE
24	YES	MEET	SWEET
25	BAD	SHY	DRY
26	ROSE	TEAR	YEAR
27	SEW	FIND	KIND
28	DENY	SLEEP	SHEEP
29	PAPER	TIE	LIE
30	WHICH	KITE	BITE
31	BRIDGE	PLACE	FACE
32	ROOSTER	GRAPE	SHAPE
33	TIME	GREEN	QUEEN
34	SURF	FIX	SIX
35	WHALE	GATE	HATE
36	SON	SNEEZE	FREEZE
37	DARK	BAKE	TAKE
38	PAINT	MATH	BATH
39	NURSE	BAR	STAR
40	CHEESE	MOUSE	HOUSE
41	FIRST	BLOW	KNOW
42	FISH	CLOWN	DOWN
43	HOME	LEARN	EARN
44	TRASH	SAME	GAME
45	GAS	WET	BET
46	WATCH	BEER	DEER
47	MOUTH	TAN	PLAN

Semantically Related Prime-Target Pairs

	Non-Rime Prime	Rime Prime	Target
1	BIRD	RAT	CAT
2	UPSET	MAD	SAD
3	FOUR	ELEVEN	SEVEN
4	ONION	TOMATO	POTATO

	Non-Rime Prime	Non-Rime Prime	Target
5	GOLF	TENNIS	SOCCER
6	YAWN	TIRED	NIGHT
7	DOCTOR	COUGH	SICK
8	PIANO	FLUTE	HARP
9	COOKIE	COW	MILK
10	MOVIE	FAMOUS	ACTOR
11	WOLF	HORSE	PIG
12	EAST	WEST	SOUTH
13	WRITE	STUDY	READ
14	FIGHT	PEACE	WAR
15	TIGER	BEAR	LION
16	SNOB	MONEY	RICH
17	MARRY	KISS	LOVE
18	ALL	SOME	HALF
19	EAGLE	TURKEY	CHICKEN
20	ASIA	JAPAN	CHINA
21	FUN	GIFT	PARTY
22	LAUGH	SMILE	HAPPY
23	SISTER	BOY	GIRL
24	STORY	PAGE	BOOK
25	COFFEE	TEA	WATER
26	HOUR	MONTH	WEEK
27	DROWN	DIVE	SWIM
28	TEACH	GRADE	CLASS
29	CORN	SALAD	CARROT
30	BLOOD	LUNG	HEART
31	ROYAL	KING	CROWN
32	ANGEL	HELL	HEAVEN
33	AUNT	UNCLE	FAMILY
34	COOL	RAIN	WIND
35	ORANGE	BANANA	APPLE
36	BALD	COMB	HAIR
37	SHELF	CHAIR	TABLE
38	EARTH	MOUNTAIN	LAND
39	EGYPT	INDIA	ITALY
40	WHO	WHEN	WHAT
41	IN	ON	UNDER
42	BORN	CRAWL	BABY
43	EAT	EGG	TOAST
44	COOK	LUNCH	HUNGRY
45	WORD	CLICK	TYPE
46	DOLL	KID	PLAY
47	STOP	FINISH	START
