# Supplementary material for: ERP Evidence for Co-Activation of English Words during Recognition of American Sign Language Signs
Source: Brain Sci. 2019 Jun 21;9(6):148. doi: 10.3390/brainsci9060148 (PMC6627215; doi:10.3390/brainsci9060148)
Supplement: Supplementary file 1 [file brainsci-09-00148-s001.zip › Table S2.pdf]

Supplementary Materials. Mean (SD) for ERP Effects by Group and Subgroup

Semantic (325-625 ms)

	Hearing Signers (N=20)		Deaf Signers (N=24)	
	Unrelated	Related	Unrelated	Related
FP1	3.94(3.70)	4.06(3.68)	1.35(4.66)	2.87(4.09)
F3	3.96(2.92)	4.45(3.42)	1.78(4.29)	3.83(3.68)
C3	4.41(2.55)	5.35(3.18)	2.85(3.47)	5.38(3.05)
P3	4.85(2.91)	6.25(3.58)	3.82(3.58)	6.70(3.56)
O1	4.15(2.72)	5.28(3.26)	3.41(3.90)	5.84(4.33)
FPz	4.43(3.61)	5.02(3.64)	1.79(4.92)	3.56(4.11)
Fz	4.26(2.92)	5.43(3.60)	2.37(4.53)	5.09(4.05)
Cz	4.27(2.84)	6.11(3.64)	2.45(4.39)	5.69(3.98)
Pz	4.60(3.19)	6.30(3.79)	3.32(3.94)	6.64(3.75)
Oz	2.44(3.25)	3.50(3.17)	1.84(3.51)	4.31(3.69)
FP2	3.93(3.22)	4.70(3.21)	1.47(4.46)	3.27(4.07)
F4	3.51(2.92)	4.80(3.32)	2.32(4.09)	4.41(3.67)
C4	4.00(3.09)	5.77(3.46)	2.40(3.06)	5.02(2.85)
P4	4.30(3.37)	5.98(3.80)	3.43(3.43)	6.25(3.26)
O2	3.52(3.91)	4.67(4.10)	3.50(3.76)	5.64(4.02)

Semantic (325-625 ms)

	Implicit Subgroup (N=14)		Explicit Subgroup (N=10)	
	Unrelated	Related	Unrelated	Related
FP1	1.00(4.48)	2.60(3.92)	1.83(5.09)	3.24(4.51)
F3	1.80(4.21)	3.76(3.57)	1.74(4.64)	3.93(4.02)
C3	2.93(3.66)	5.43(3.24)	2.74(3.38)	5.31(2.93)
P3	3.50(3.67)	6.55(4.10)	4.26(3.59)	6.90(2.84)
O1	2.86(3.53)	5.43(4.50)	4.18(4.46)	6.41(4.25)
FPz	1.53(4.46)	3.58(3.96)	2.15(5.75)	3.52(4.54)
Fz	2.30(4.40)	5.12(3.85)	2.48(4.95)	5.03(4.54)
Cz	2.47(4.56)	6.00(4.40)	2.43(4.37)	5.24(3.48)
Pz	2.87(4.19)	6.55(4.25)	3.95(3.69)	6.78(3.14)
Oz	1.26(3.59)	4.08(4.32)	2.65(3.41)	4.64(2.78)
FP2	1.21(3.88)	3.13(3.86)	1.85(5.37)	3.47(4.54)
F4	2.07(3.79)	4.30(3.79)	2.66(4.68)	4.57(3.70)
C4	2.18(2.99)	5.18(3.39)	2.70(3.30)	4.79(2.02)
P4	3.09(3.96)	6.17(3.89)	3.92(2.66)	6.36(2.29)
O2	2.61(3.67)	5.04(4.33)	4.75(3.70)	6.47(3.60)

Rime (325-625 ms)

	Hearing Signers (N=20)		Deaf Signers (N=24)	
	Unrelated	Related	Unrelated	Related
FP1	3.84(3.94)	4.42(3.75)	1.92(4.54)	0.77(4.71)
F3	4.30(3.16)	3.90(3.38)	2.21(4.52)	1.19(4.35)
C3	4.53(2.64)	4.53(2.87)	3.24(3.94)	2.35(3.69)
P3	4.70(2.81)	5.00(3.28)	4.06(3.81)	3.37(4.00)
O1	3.85(2.46)	4.23(3.12)	3.44(4.00)	3.16(4.11)
FPz	4.19(3.94)	5.00(3.63)	2.52(5.06)	0.91(4.76)
Fz	4.32(3.16)	4.62(3.26)	2.61(4.77)	1.92(4.62)
Cz	4.12(2.86)	4.75(3.43)	2.65(4.93)	2.15(4.61)
Pz	4.22(3.34)	4.96(3.71)	3.47(4.23)	2.92(4.37)
Oz	2.04(3.21)	2.61(3.62)	1.81(3.42)	1.66(4.08)
FP2	3.71(3.44)	4.59(3.25)	1.78(4.70)	1.04(4.25)
F4	3.52(2.92)	3.95(3.34)	2.48(4.28)	2.08(4.07)
C4	3.69(3.19)	4.54(3.72)	2.42(3.49)	2.24(3.20)
P4	3.82(3.45)	4.67(3.99)	3.55(3.89)	3.09(3.66)
O2	2.87(3.97)	3.90(4.29)	3.23(3.69)	3.32(3.95)

Rime (325-625 ms)

	Implicit Subgroup (N=14)		Explicit Subgroup (N=10)	
	Unrelated	Related	Unrelated	Related
FP1	2.10(3.73)	0.77(4.33)	2.21(4.77)	1.51(5.27)
F3	2.73(3.39)	1.49(3.78)	1.80(5.03)	1.40(4.81)
C3	3.87(3.54)	2.91(3.74)	2.69(4.21)	2.22(3.74)
P3	4.39(3.99)	3.83(3.90)	4.21(3.82)	3.51(4.14)
O1	3.47(3.67)	3.36(3.68)	4.20(4.56)	3.49(4.50)
FPz	2.65(3.61)	0.89(4.20)	2.59(6.08)	1.68(5.43)
Fz	3.13(3.60)	2.09(4.34)	2.09(5.44)	2.47(5.08)
Cz	3.44(4.42)	2.70(4.83)	1.99(5.37)	2.20(4.66)
Pz	3.93(4.37)	3.23(4.51)	3.64(4.30)	3.32(4.15)
Oz	2.15(3.66)	1.67(3.61)	2.45(3.14)	2.18(4.01)
FP2	2.06(3.37)	0.95(3.66)	1.74(5.54)	1.72(5.25)
F4	2.78(3.05)	1.90(3.69)	2.24(4.85)	2.93(4.63)
C4	2.99(2.86)	2.44(2.96)	2.18(3.88)	2.64(3.46)
P4	4.26(4.41)	3.38(3.81)	3.52(3.02)	3.38(3.00)
O2	3.35(3.69)	3.10(3.80)	4.27(3.23)	4.12(3.88)

Rime (700-900 ms)

	Hearing Signers (N=20)		Deaf Signers (N=24)	
	Unrelated	Related	Unrelated	Related
FP1	4.02(4.60)	5.84(3.77)	2.69(5.19)	1.63(5.91)
F3	5.30(4.54)	5.97(3.92)	3.16(4.29)	2.12(4.52)
C3	5.90(4.07)	7.21(3.87)	4.71(4.42)	3.75(3.85)
P3	6.70(3.65)	8.82(4.48)	6.32(4.91)	5.63(4.49)
O1	4.25(2.18)	6.39(3.80)	4.51(4.03)	4.59(4.34)
FPz	5.12(4.68)	7.98(3.63)	3.65(5.91)	2.25(6.06)
Fz	5.49(4.95)	7.97(3.92)	4.05(4.26)	3.34(4.04)
Cz	5.89(5.14)	9.08(5.07)	5.66(4.83)	5.11(4.40)
Pz	6.82(4.22)	9.96(5.17)	7.90(5.35)	7.32(4.96)
Oz	3.18(3.12)	5.98(4.16)	4.74(3.72)	4.75(4.05)
FP2	4.25(4.31)	6.80(3.95)	3.11(5.38)	2.46(5.28)
F4	3.94(4.73)	6.42(4.48)	3.97(3.81)	3.49(3.79)
C4	4.76(4.88)	7.55(4.56)	4.91(3.75)	4.80(3.56)
P4	5.09(4.39)	8.18(4.91)	6.37(4.63)	6.08(4.17)
O2	2.40(3.65)	5.41(3.99)	4.46(4.22)	4.81(4.32)

Rime (700-900 ms)

	Implicit Subgroup (N=14)		Explicit Subgroup (N=10)	
	Unrelated	Related	Unrelated	Related
FP1	3.51(5.39)	1.84(6.27)	1.54(4.94)	1.35(5.67)
F3	4.23(4.74)	2.82(4.66)	1.65(3.20)	1.14(4.36)
C3	5.64(4.59)	4.15(3.80)	3.40(4.03)	3.18(4.04)
P3	6.33(4.98)	5.11(4.59)	6.30(5.08)	6.35(4.49)
O1	3.91(4.53)	3.57(4.45)	5.36(3.23)	6.03(3.94)
FPz	4.43(5.80)	2.05(5.99)	2.55(6.20)	2.54(6.48)
Fz	4.95(4.71)	3.47(3.95)	2.79(3.35)	3.15(4.37)
Cz	6.40(4.75)	5.07(3.70)	4.62(4.99)	5.18(5.44)
Pz	7.89(4.98)	6.64(4.44)	7.92(6.11)	8.27(5.73)
Oz	4.31(4.17)	3.90(4.14)	5.35(3.11)	5.94(3.81)
FP2	3.61(5.72)	2.33(5.05)	2.41(5.08)	2.64(5.87)
F4	4.38(4.35)	3.16(3.69)	3.38(3.02)	3.96(4.09)
C4	4.93(4.08)	4.09(3.04)	4.89(3.44)	5.80(4.13)
P4	6.24(5.33)	5.25(4.17)	6.55(3.70)	7.24(4.08)
O2	3.58(4.89)	3.70(4.38)	5.68(2.86)	6.36(3.93)
