# Supplementary material for: ERP Evidence for Co-Activation of English Words during Recognition of American Sign Language Signs
Source: Brain Sci. 2019 Jun 21;9(6):148. doi: 10.3390/brainsci9060148 (PMC6627215; doi:10.3390/brainsci9060148)

Supplementary Materials. Scatterplots of significant correlations between ERP effects and language measures at representative sites

Semantic (325-625 ms)

Subject	ASL Production	Difference at FP1
s1	0.83	-0.98
s2	0.87	0.56
s3	0.77	0.71
s4	0.8	-3.78
s5	0.83	1.4
s6	0.83	-1.53
s7	0.83	1.28
s8	0.83	0.72
s9	0.9	1.76
s10	0.8	-0.45
s11	0.77	0.16
s12	0.93	2.57
s13	0.77	-2.48
s14	0.87	0.16
s15	0.8	1.13
s16	0.77	0.37
s17	0.83	2.19
s18	0.83	-1.72
s19	0.7	-4.2
s20	0.83	-0.24

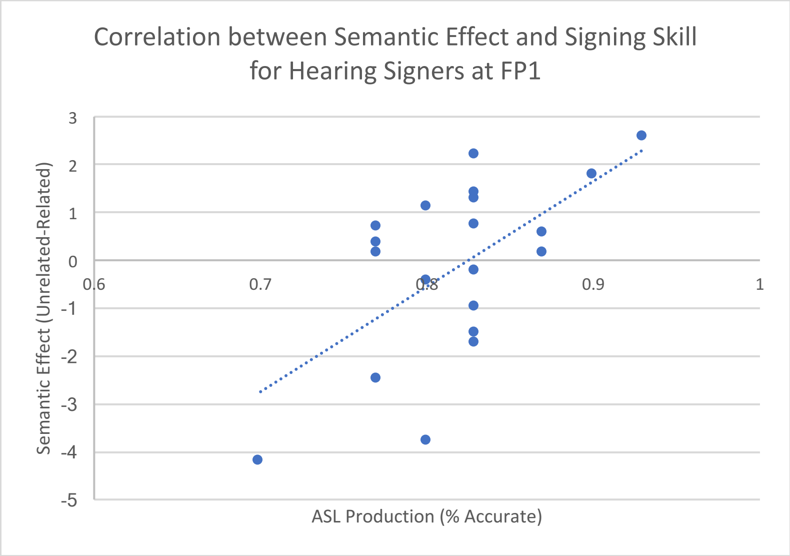

Rime (325-625 ms)

Subject	ASL Comprehension	Difference at Pz
s1	8	-0.12
s2	18	-3.68
s3	17	-4.35
s4	14	0.02
s5	5	3.4
s6	16	-0.9
s7	14	-1.48
s8	10	-0.05
s9	17	0.05
s10	23	-5.17
s11	11	-0.17
s12	22	-0.37
s13	11	-0.52
s14	24	-4.56
s15	20	-2.06
s16	17	-1.3
s17	15	-0.24
s18	12	3.92
s19	11	0.69
s20	11	1.98

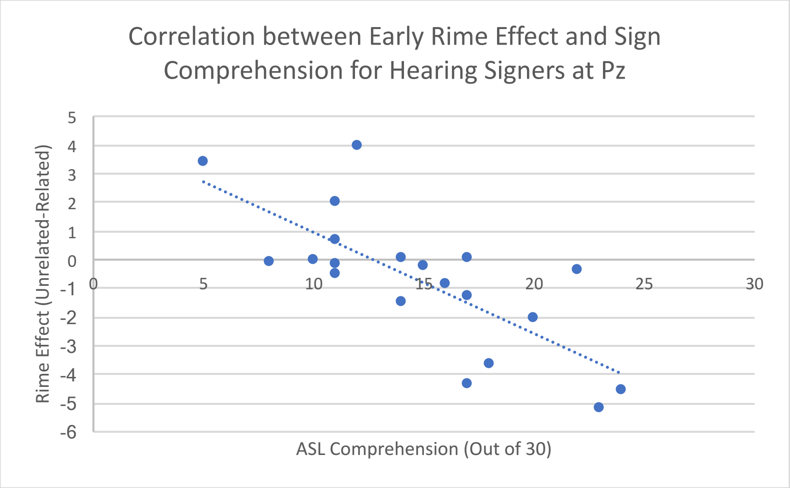

Rime (700-900 ms)

Subject	ASL Comprehension	Difference at Pz
s1	8	-5.92
s2	18	-7.36
s3	17	-8.79
s4	14	-0.01
s5	5	3.7
s6	16	-6.59
s7	14	-5.04
s8	10	2.58
s9	17	1.29
s10	23	-7.51
s11	11	0.16
s12	22	-5.73
s13	11	-6.46
s14	24	-9.21
s15	20	-9.5
s16	17	-1.3
s17	15	2.1
s18	12	0.76
s19	11	-2.08
s20	11	2.18

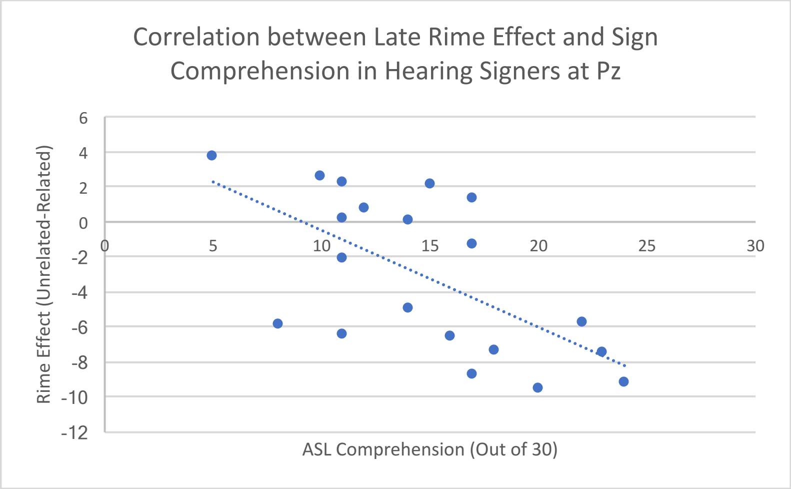

Rime (700-900 ms)

Subject	English Spelling	Difference at P3
s1	78	-3.38
s2	81	-5.85
s3	82	-6.59
s4	73	2.95
s5	79	3.27
s6	83	-8.22
s7	80	-5.02
s8	73	1.1
s9	79	1.06
s10	80	-1.4
s11	76	0.36
s12	85	-5.26
s13	75	-4.97
s14	83	-6.87
s15	83	-6.38
s16	79	0.12
s17	80	1.16
s18	80	1.3
s19	78	-1.91
s20	73	2.26

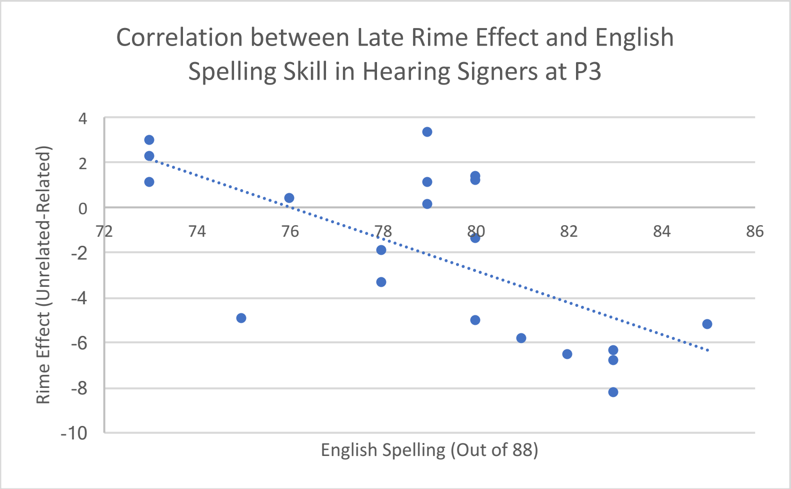

Supplement: Supplementary file 1 [file brainsci-09-00148-s001.zip › Table S3.pdf]
