# Supplementary material for: ERP Evidence for Co-Activation of English Words during Recognition of American Sign Language Signs
Source: Brain Sci. 2019 Jun 21;9(6):148. doi: 10.3390/brainsci9060148 (PMC6627215; doi:10.3390/brainsci9060148)
Supplement: Supplementary file 1 [file brainsci-09-00148-s001.zip › Table S4.pdf]

Supplementary Materials. Pearson Correlations with FDR-Adjusted *p* Values

Hearing Signers

			Prefrontal			Frontal			Central			Parietal			Occipital		
			FP1	FPz	FP2	F3	Fz	F4	C3	Cz	C4	P3	Pz	P4	O1	Oz	O2
Semantic (325-625 ms)		*r*	0.431	0.328	0.371	0.244	0.117	0.174	0.148	0.131	0.143	0.275	0.252	0.217	0.411	0.381	0.311
	English Spelling	*p*	0.401	0.453	0.401	0.498	0.624	0.624	0.624	0.624	0.624	0.498	0.498	0.539	0.401	0.401	0.453
		r	0.284	0.289	0.193	0.393	0.246	0.212	0.363	0.306	0.216	0.443	0.380	0.361	0.502	0.460	0.438
	ASL Comprehension	*p*	0.305	0.305	0.415	0.221	0.370	0.396	0.221	0.305	0.396	0.203	0.221	0.221	0.203	0.203	0.203
		r	0.608	0.601	0.588	0.542	0.548	0.508	0.444	0.454	0.431	0.336	0.348	0.289	0.410	0.368	0.272
	ASL Production	*p*	0.030	0.030	0.030	0.042	0.042	0.055	0.094	0.094	0.097	0.171	0.166	0.231	0.108	0.150	0.246
Rime (325-625 ms)		*r*	-0.091	-0.163	-0.284	-0.201	-0.148	-0.314	-0.343	-0.334	-0.411	-0.468	-0.406	-0.426	-0.342	-0.384	-0.357
	English Spelling	*p*	0.704	0.569	0.305	0.494	0.570	0.267	0.252	0.252	0.252	0.252	0.252	0.252	0.252	0.252	0.252
		r	-0.311	-0.409	-0.492	-0.109	-0.331	-0.444	-0.240	-0.492	-0.619	-0.543	-0.745	-0.681	-0.502	-0.678	-0.575
	ASL Comprehension	*p*	0.209	0.100	0.047	0.646	0.193	0.075	0.330	0.047	0.015	0.033	0.000	0.005	0.047	0.005	0.024
		r	0.221	0.107	0.029	0.296	0.330	0.171	0.127	0.123	0.008	-0.125	-0.005	-0.036	-0.154	-0.202	-0.291
	ASL Production	*p*	0.889	0.889	0.982	0.889	0.889	0.889	0.889	0.889	0.982	0.889	0.982	0.982	0.889	0.889	0.889
Rime (700-900 ms)		*r*	-0.048	-0.232	-0.318	-0.341	-0.378	-0.436	-0.520	-0.554	-0.510	-0.662	-0.604	-0.535	-0.635	-0.594	-0.554
	English Spelling	*p*	0.840	0.348	0.198	0.176	0.136	0.083	0.036	0.028	0.037	0.015	0.023	0.032	0.023	0.023	0.028
		r	-0.065	-0.183	-0.227	-0.155	-0.313	-0.348	-0.317	-0.477	-0.468	-0.514	-0.625	-0.512	-0.568	-0.595	-0.544
	ASL Comprehension	*p*	0.785	0.507	0.419	0.551	0.244	0.220	0.244	0.071	0.071	0.053	0.045	0.053	0.045	0.045	0.049
		r	0.134	0.089	0.049	0.245	0.250	0.222	0.046	0.108	0.056	-0.095	-0.009	0.009	-0.103	-0.130	-0.179
	ASL Production	*p*	0.970	0.970	0.970	0.970	0.970	0.970	0.970	0.970	0.970	0.970	0.970	0.970	0.970	0.970	0.970

Deaf Signers

			Prefrontal			Frontal			Central			Parietal			Occipital		
			FP1	FPz	FP2	F3	Fz	F4	C3	Cz	C4	P3	Pz	P4	O1	Oz	O2
Semantic (325-625 m		*r*	-0.139	-0.038	-0.090	-0.209	-0.200	-0.071	-0.297	-0.121	0.044	-0.207	-0.079	-0.020	-0.064	-0.060	-0.061
	English Spelling	*p*	0.923	0.923	0.923	0.923	0.923	0.923	0.923	0.923	0.923	0.923	0.923	0.924	0.923	0.923	0.923
		r	-0.020	-0.122	-0.133	-0.058	-0.066	-0.055	-0.041	-0.046	-0.061	0.089	0.083	-0.005	0.163	0.227	0.180
	ASL Comprehension	*p*	0.983	0.980	0.980	0.980	0.980	0.980	0.980	0.980	0.980	0.980	0.980	0.983	0.980	0.980	0.980
		r	0.161	0.200	0.191	0.074	0.084	0.150	-0.116	0.062	0.191	-0.041	0.053	0.080	0.027	0.143	0.133
	ASL Production	*p*	0.902	0.902	0.902	0.902	0.902	0.902	0.902	0.902	0.902	0.902	0.902	0.902	0.902	0.902	0.902
Rime (325-625 ms)		*r*	-0.409	-0.252	-0.205	-0.373	-0.350	-0.268	-0.225	-0.244	-0.215	-0.103	-0.204	-0.130	-0.048	-0.096	-0.130
	English Spelling	*p*	0.470	0.510	0.510	0.470	0.470	0.510	0.510	0.510	0.510	0.703	0.510	0.681	0.825	0.703	0.681
		r	-0.101	-0.122	-0.011	-0.177	-0.244	-0.138	-0.230	-0.168	-0.109	-0.066	-0.168	-0.058	0.005	-0.095	-0.049
	ASL Comprehension	*p*	0.946	0.946	0.983	0.946	0.946	0.946	0.946	0.946	0.946	0.946	0.946	0.946	0.983	0.946	0.946
		r	-0.146	-0.047	-0.028	-0.157	-0.173	-0.114	-0.118	-0.010	0.072	0.074	0.087	0.142	0.168	0.184	0.242
	ASL Production	*p*	0.924	0.954	0.961	0.924	0.924	0.924	0.924	0.962	0.924	0.924	0.924	0.924	0.924	0.924	0.924
Rime (700-900 ms)		*r*	-0.078	-0.113	-0.042	-0.202	-0.291	-0.144	-0.369	-0.417	-0.357	-0.443	-0.464	-0.407	-0.420	-0.402	-0.424
	English Spelling	*p*	0.769	0.691	0.846	0.468	0.251	0.626	0.143	0.109	0.145	0.109	0.109	0.109	0.109	0.109	0.109
		r	0.039	-0.074	0.018	-0.004	-0.135	-0.035	-0.184	-0.177	-0.104	-0.265	-0.310	-0.168	-0.236	-0.229	-0.147
	ASL Comprehension	*p*	0.984	0.984	0.984	0.984	0.883	0.984	0.883	0.883	0.945	0.883	0.883	0.883	0.883	0.883	0.883
		r	-0.075	-0.184	-0.211	-0.161	-0.312	-0.305	-0.299	-0.318	-0.262	-0.221	-0.237	-0.195	0.008	0.005	0.001
	ASL Production	*p*	0.908	0.585	0.585	0.618	0.585	0.585	0.585	0.585	0.585	0.585	0.585	0.585	0.997	0.997	0.997
